# Modeling potential distribution of newly recorded ant, *Brachyponera nigrita* using Maxent under climate change in Pothwar region, Pakistan

**DOI:** 10.1371/journal.pone.0262451

**Published:** 2022-01-19

**Authors:** Ammara Gull E. Fareen, Tariq Mahmood, Imran Bodlah, Audil Rashid, Azeem Khalid, Shahid Mahmood

**Affiliations:** 1 Institute of Soil and Environmental Sciences, Pir Mehr Ali Shah Arid Agriculture University, Rawalpindi, Pakistan; 2 Department of Environmental Sciences, University of Narowal, Narowal, Pakistan; 3 Insect Biodiversity and Conservation Group, Department of Entomology, Pir Mehr Ali Shah Arid Agriculture University, Rawalpindi, Pakistan; 4 Department of Botany, University of Gujrat, Gujrat, Pakistan; Ghazi University, PAKISTAN

## Abstract

Climate change has been discussed as to exert shifts in geographical range of plants, animals or insect species by increasing, reducing or shifting its appropriate climatic habitat. Globally, Pakistan has been ranked at 5^th^ position on the list of countries most vulnerable to climate change in 2020. Climate change has resulted in the losses of biodiversity and alteration in ecosystem as a result of depletion of natural habitats of species in Pakistan as well as in the world. Ants have been regarded as indicators of environmental change and ecosystem processes. *Brachyponera nigrita* (Emery, 1895) was reported for the first time from Pakistan (Pothwar region). Objective of our studies was to model geographic distribution of newly recorded ant species, *B*. *nigrita* based on two representative concentration pathways (RCP) (RCP4.5 and RCP8.5) for 2050s using maximum entropy model (Maxent) in Pakistan. In modeling procedure, 21occurrence records and 8 variables namely Bio4 (Temperature seasonality), Bio8 (Mean temperature of wettest quarter), Bio10 (Mean temperature of warmest quarter), Bio12 (Annual precipitation), Bio13 (Precipitation of wettest month), Bio15 (Precipitation seasonality), Bio17 (Precipitation of driest quarter) and Bio18 (Precipitation of warmest quarter) were used to determine the current and future distributions. Performance of the model was evaluated using AUC (area under curves) values, partial ROC, omission rates (E = 5%) and AICc (Model complexity).The results showed the average AUC value of the model was 0.930, which indicated that the accuracy of the model was excellent. The jackknife test also showed that Bio4, Bio18, Bio17 and Bio15 contributed 98% for the prediction of potential distribution of the species as compared to all other variables. Maxent results indicated that distribution area of *B*. *nigrita* under future predicted bioclimatics 2050 (RCP 4.5 and RCP8.5) would be increased in various localities of Pakistan as compared to its current distribution. In Pothwar region, moderately suitable and highly suitable areas of this species would increase by 505.932321km^2^and 572.118421km^2^as compared to current distribution under 2050 (RCP 4.5), while under 2050 (RCP 8.5), there would be an increase of 6427.2576km^2^and 3765.140493km^2^ respectively in moderately suitable and highly suitable areas of *B*. *nigrita*. This species was associated with termites, collembolans and larval stages of different insects. White eggs, creamy white pupae and many workers of this species were observed in a variety of habitats. Unknown nesting ecology, species identification characters supported with micrographs has been given which will help researchers for further ecological studies.

## Introduction

Climate Change has been recognized as obvious and emerging global phenomenon. It’s adverse effects on biological entities have also been reported throughout the world [1–6]. The Fifth Assessment Report recently presented by IPCC (Intergovernmental Panel on Climate Change) emphasized on the reduction of anthropogenic climate change, produced by greenhouse gas emission [7]. The main reason for undertaking such types of protocol is to protect biodiversity from the impacts of global warming [8–10]. Impacts of climate change has been reported for humans as well as biodiversity, including disease [11, 12] and pest outbreaks [13], shift in species distribution, occurrence and Phenology [14]. The condition is predicted to become spectacular as the magnitude of environmental change accelerates [15]. This situation is expected to be more adverse in developing nations [16]. Climate change has direct effect on the survival, distribution, development [17, 18] and abundance of living organisms [19–21]. Arthropods are ideal organisms for determining biodiversity. Among insects, members of family Formicidae are significant target group to examine biodiversity [22]. Ants are abundant, ubiquitous and also recognized to be very sensitive to change in temperature and humidity as they affect survival and foraging activity [23].

The taxonomic status of *Brachyponera* Emery has complex history, is presently synonymized as Pachycondyla, very large genus [24, 25]. It comprises species from Oriental, Australian and Ethiopian regions. Oriental region includes a few species from tropics as limited taxonomic work has been done there. Genus *Brachyponera* is reasonable genus with 19 valid species and 5 subspecies, remarkable among other Ponerines with distinct size and dimorphism among workers and queens. Some species exhibit invasive behavior [26]. Workers of genus *Brachyponera* are small, solitary, scavengers and predatory in behavior. Most of the species construct their nests in rotten wood or soil [27]. This genus is distinct from other Ponerinae species due to the presence of pests and invasive species having dangerous sting.

ENM (Ecological niche modeling), similar mechanisms of habitat suitability and species distribution modeling have gained recognition as they enable estimation of possible geographic species distribution under climate change scenarios [28]. The methods provide information about the environmental variables related to the ecological niche inhibition on species related to future distribution approximated by climate models [29]. These models result in coarse resolution correlative models of ecological niche that predict the potential geographical distribution of species under current and future climatic conditions. Maxent has been used in a number of studies involving prediction of different ant species in different parts of the world. Senula et al. [30] applied this model to predict the habitat suitability of ants belonging to the genus *Trachymyrmex* and *Mycetomoellerius* in the United States. Song et al. [31] modeled *Solenopsis invicta* (Hymenoptera: Formicidae) using maxent for current and future projected climatic conditions in China. Koch et al. [32] implemented maxent and determined areas of highest suitability for *Gracilidris pombero* Wild & Cuezzo, 2006.

A limited work has been done on the taxonomy of ants in Pakistan. Recently, Rasheed et al. [33]. recorded 103 ant species from Pakistan. Prior to the present studies, no species of genus *Brachyponera* has been reported from Pakistan. To the best of our knowledge, to date no species of insects particularly ants have been modeled for projected climatic conditions in Pakistan. Changing climatic conditions would have numerous impacts on the ecological interactions of the species specifically which are affected by environmental factors. Keeping in view this situation, newly recorded ant species, *B*. *nigrita* was modeled for current and future climatic conditions in Pakistan.

## Materials and methods

### Ant’s collection and identification

Ants were collected from different localities and diverse habitats like leaf litter, under stone, tree barks, soil surface, crop plants, grasses, ornamentals etc. Nests of ants were photographed with the help of professional camera. Ants were collected by using aspirator, net sweeping, handpicking etc and killed using killing bottle (Potassium Cyanide). The killed specimens were preserved in 75% alcohol. Preserved specimens were identified using stage microscope and identification keys by Bingham [34] and Bharti [35]. Micrographs of identified specimens were prepared using tri-nocular microscope (Leica MS5 microscope) attached with camera (Amscope 18 megapixel).

### Survey and data collection

**Fig 1:**
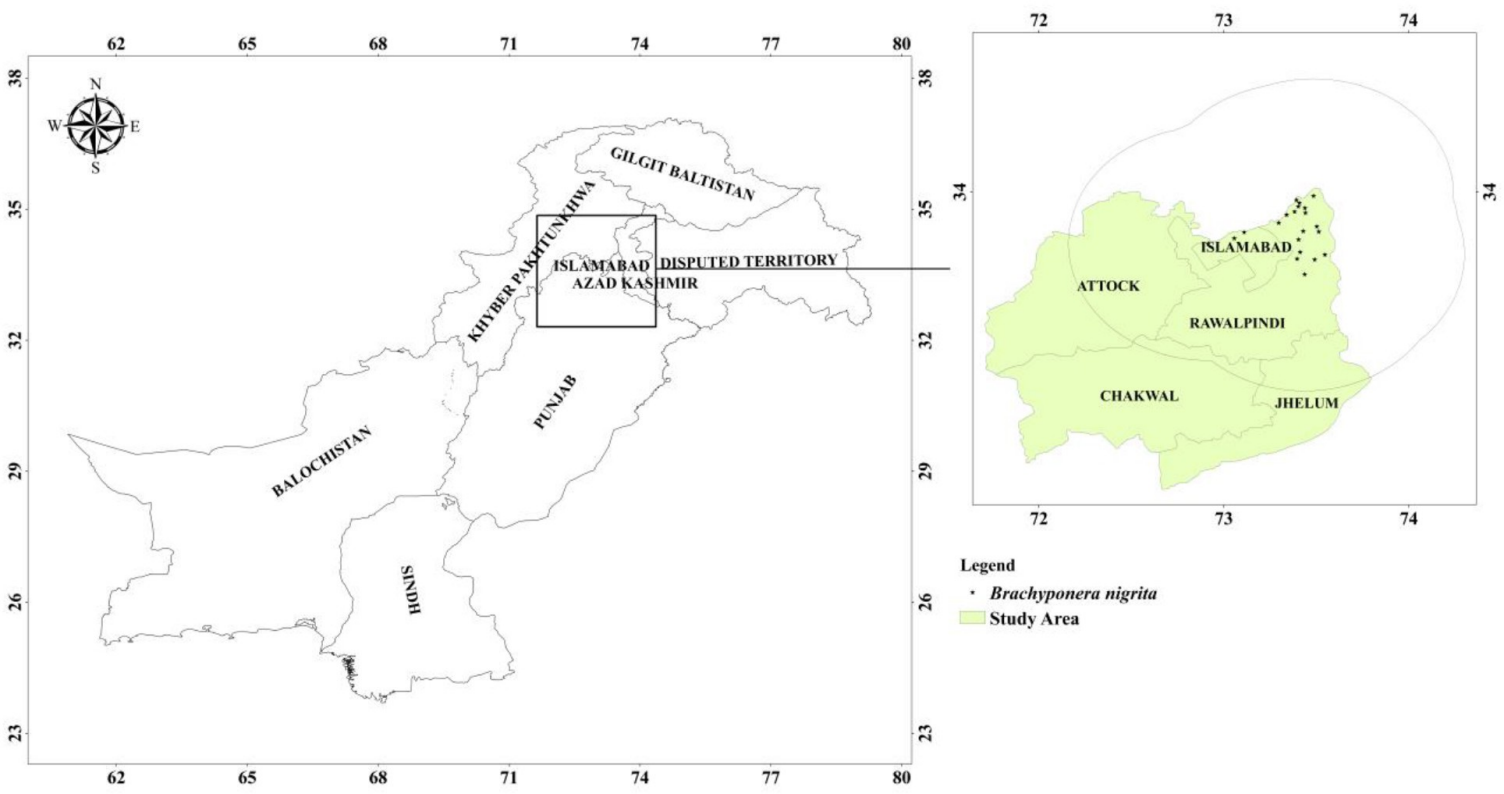
Occurrence points of the species along with buffer of 70 kilometers (M).

About thirty multisite surveys were conducted in four districts of Pothwar region including Capital territory (Islamabad) for the collection of ants in various habitats ranging from grasslands, mountains, forest areas etc. Every sampling site was surveyed 500 ×500 metrs for collection of ants from trees, flowers, ant nests, plants etc. As a result of these surveys, *B*. *nigrita* was collected from 29 sites. Data was recorded in the form of geographical coordinates of *B*. *nigrita* occurrences with the help of GPS device (Garmin e Trix 10). Data collection technique used in present study was the presence-only data collection method as used in various studies for species distribution modeling [36–39]. The R package (The spThin) was used for spatial thinning of occurrence points [40]. In order to reduce biases related to spatial autocorrelation, occurrence data were rarefied in a pattern that a pair of points was not closer than three kilometers. This process resulted in a total of 21 occurrence points of *B*. *nigrita*. Out of these 17 occurrences were processed in ArcGIS software (Version 10.4.1) and a buffer 70 km was made around all occurrence points for setting model calibration area (M) (Fig 1). M (Calibration area) area is an area which is considered an accessible area for a species over time as described by Barve et al. [41]. Remaining 4 occurrences were used as independent data.

### Bio-climatic predictors

A set of 19 bio-climatic predictor variables were obtained from official source (WorldClim website; Global Climate Data) for *B*. *nigrita* species distribution and suitability analysis. They were raster layers with a spatial resolution of 30 arc seconds. These variables are often used in many ecological and biogeographic studies for modeling species distribution, niche modeling and habitat suitability of different organisms [42, 43]. They are availed from various sources like WorldClim database, CliMond database etc.

These bioclimatic variables have been approved to indicate general patterns of precipitation and temperature, extremity and seasonality of temperature. These layers were converted according to model calibration area (M) by extract by mask function using ArcGIS software. These were then converted to Ascii format. The multicollinearity between 19 bio-climatic predictors was determined using Pearson correlation coefficient (r) with help of ENM Tools software v1.3.1.,cross-correlation value set at (r) ≥ 0.90 in order to remove strongly correlated predictors [44–47]. (Table 1). The 8 variables were selected based on their potential relevance to explain the distribution of species. The Maxent (Version 3.3.4k) [48] was run along with occurrence points, the results of jackknife test were used to determine dominant environmental variables that determined the species potential distribution. After screening of climatic variables, finally eight variables selected for the model run; Bio4 (Temperature seasonality), Bio8 (Mean temperature of wettest quarter), Bio10 (Mean temperature of warmest quarter), Bio12 (Annual precipitation), Bio13 (Precipitation of wettest month), Bio15 (Precipitation seasonality), Bio17 (Precipitation of driest quarter) and Bio18 (Precipitation of warmest quarter).

**Table 1:** Correlation matrix of predictor variables.

Species	bio_1.asc	bio_2.asc	bio_3.asc	bio_4.asc	bio_5.asc	bio_6.asc	bio_7.asc	bio_8.asc	bio_9.asc	bio_10.asc	bio_11.asc	bio_12.asc	bio_13.asc	bio_14.asc	bio_15.asc	bio_16.asc	bio_17.asc	bio_18.asc	bio_19.asc
**bio_1.asc**	1	0.957246	0.953287	0.384191	0.990836	0.94362	0.859681	0.878374	0.997302	0.993847	0.9935	-0.30527	0.300932	-0.64153	0.798555	0.226804	-0.63148	0.085732	-0.49673
**bio_2.asc**	1	1	0.890625	0.606026	0.984399	0.815229	0.962121	0.873177	0.940801	0.981121	0.923007	-0.36748	0.250226	-0.70018	0.817287	0.166172	-0.68987	0.051582	-0.53249
**bio_3.asc**	1	1	1	0.221157	0.925385	0.955411	0.737402	0.76583	0.957791	0.933902	0.965517	-0.23629	0.303783	-0.51437	0.688782	0.240079	-0.50643	0.092852	-0.3903
**bio_4.asc**	1	1	1	1	0.488273	0.067048	0.775263	0.45396	0.326816	0.477218	0.27786	-0.48547	-0.11514	-0.65065	0.500052	-0.18125	-0.65471	-0.22767	-0.54805
**bio_5.asc**	1	1	1	1	1	0.894542	0.918707	0.894788	0.983052	0.99877	0.97204	-0.33348	0.280826	-0.6723	0.810563	0.200858	-0.66154	0.079302	-0.51194
**bio_6.asc**	1	1	1	1	1	1	0.645291	0.774052	0.959123	0.904501	0.973403	-0.17545	0.33766	-0.46497	0.663065	0.28276	-0.45498	0.134383	-0.35296
**bio_7.asc**	1	1	1	1	1	1	1	0.84535	0.832679	0.907806	0.801243	-0.41493	0.181614	-0.73819	0.799467	0.093448	-0.72863	0.016798	-0.5631
**bio_8.asc**	1	1	1	1	1	1	1	1	0.875962	0.88685	0.858891	-0.11999	0.431774	-0.52562	0.811055	0.355322	-0.50208	0.288361	-0.33585
**bio_9.asc**	1	1	1	1	1	1	1	1	1	0.985762	0.99793	-0.26965	0.324522	-0.60626	0.784826	0.251625	-0.59498	0.116282	-0.45776
**bio_10.asc**	1	1	1	1	1	1	1	1	1	1	0.976322	-0.34085	0.270252	-0.67362	0.802748	0.191677	-0.6638	0.060902	-0.51891
**bio_11.asc**	1	1	1	1	1	1	1	1	1	1	1	-0.25655	0.326419	-0.58417	0.765034	0.257342	-0.57299	0.119689	-0.44445
**bio_12.asc**	1	1	1	1	1	1	1	1	1	1	1	1	0.762207	0.853077	-0.11039	0.808736	0.870595	0.826082	0.913756
**bio_13.asc**	1	1	1	1	1	1	1	1	1	1	1	1	1	0.339966	0.542342	0.991478	0.361765	0.865709	0.488258
**bio_14.asc**	1	1	1	1	1	1	1	1	1	1	1	1	1	1	-0.58915	0.409927	0.992418	0.517996	0.951045
**bio_15.asc**	1	1	1	1	1	1	1	1	1	1	1	1	1	1	1	0.477228	-0.57148	0.271254	-0.43994
**bio_16.asc**	1	1	1	1	1	1	1	1	1	1	1	1	1	1	1	1	0.434775	0.872771	0.540834
**bio_17.asc**	1	1	1	1	1	1	1	1	1	1	1	1	1	1	1	1	1	0.551437	0.963581
**bio_18.asc**	1	1	1	1	1	1	1	1	1	1	1	1	1	1	1	1	1	1	0.659795
**bio_19.asc**	1	1	1	1	1	1	1	1	1	1	1	1	1	1	1	1	1	1	1

### Settings of the Maxent model using R package Kuenm

Maxent was run using *R package Kuenm* [49] with the aim to produce preliminary models with occurrence data and environmental predictors. Maxent has been used for modeling different species in various parts of the world and is a renowned tool implemented for species distribution modeling. *R package Kuenm* automates ENM processes; help the users to manage model complexity and testing of data with a number of parameters. Occurrence data (17 points) was divided randomly into 5 testing and 12 training for model calibration and internal testing. We already set aside 4 occurrences as independent data for independent model testing. Three sets of selected 8 variables were also randomly prepared as Set1 (Bio4, Bio10, Bio13 and Bio18), Set 2 (Bio8, Bio12, Bio15 and Bio17) and Set 3 (combination of all eight selected variables).These three sets were named as M variables. Kuenm_ceval function of *Kuenm* was used for the preparation of preliminary models. Calibration of these models was done for the evaluation of best possible combination of parameters to be selected for running Maxent in order to prepare best model for our species. Candidate models were prepared using all combination of 17 value of regularization\ multiplier and 29 feature class combinations as used by Cobos et al. [49]. Later on evaluation of these models was carried out on the basis of partial ROC (with 500 iterations), omission rates (E = 5%) and AICc (Model complexity).Then from these model sets, best model was selected with delta AICc values ≤ 2. *kuenm_ceval* function was used for evaluation and selection of best candidate model. Similarly *kuenm_mod* and *kuenm_feval* function of the package were used for evaluation and selection of final models. Maxent was run with 500 iterations, 10 random replicate analyses, all other options were of default settings of *kuenm package*.

### Transfer of calibration area model results

After the selection of best model for calibration area, model results were transferred to Pothwar and Pakistan region for current and future scenarios with RCPs 4.5 and 8.5 for the year of 2050 based on 5th assessment report of IPCC. For this purpose parallel bioclimatic data layers were obtained for three GCM (HadGEM2-AO, HadGEM2-ES and BCC-CSM1-1).These predictor layers were prepared with same procedure as given above as G variables and model results were projected to two regions and two future scenarios. Finally we explored 3 GCMs 1 time period 2 RCPs = 6 future transfers of models. Results of Maxent were imported in ArcGIS software and further processed for the preparation of maps. Raster layers (Pothwar region only) were projected to WGS 1984 UTM zone 42 North and reclassified for the measurement of geographical area of the species. Area calculation was performed only for Pothwar region (area of interest).

### Development of binary models

Binary models were developed on the basis of relationship of calibration points and raw model output of maxent. Raw outputs were imported to ArcGIS software along with calibration points. Then with extract values to point’s function of ArcGIS, we decided threshold values; the highest thresholds corresponding to 0%, 5%, and 10% omission error rates as used by Ashraf et al. [50]. Change in high, moderate, and low suitability areas was calculated with same criteria for current and all future scenarios. The set threshold were as follows threshold values from0–0.18586 (E = 0–5%) as low suitability area, 0.18586–0.22912 threshold value (E = 5–10%) as moderate suitability area and above 0.22912 threshold value as highly suitable area. Future-transfer maps were also prepared with the same criteria as done for present day.

## Results

### Identification characters of *Brachyponera nigrita* (Emery, 1895) (Fig 2)

**Fig 2:**
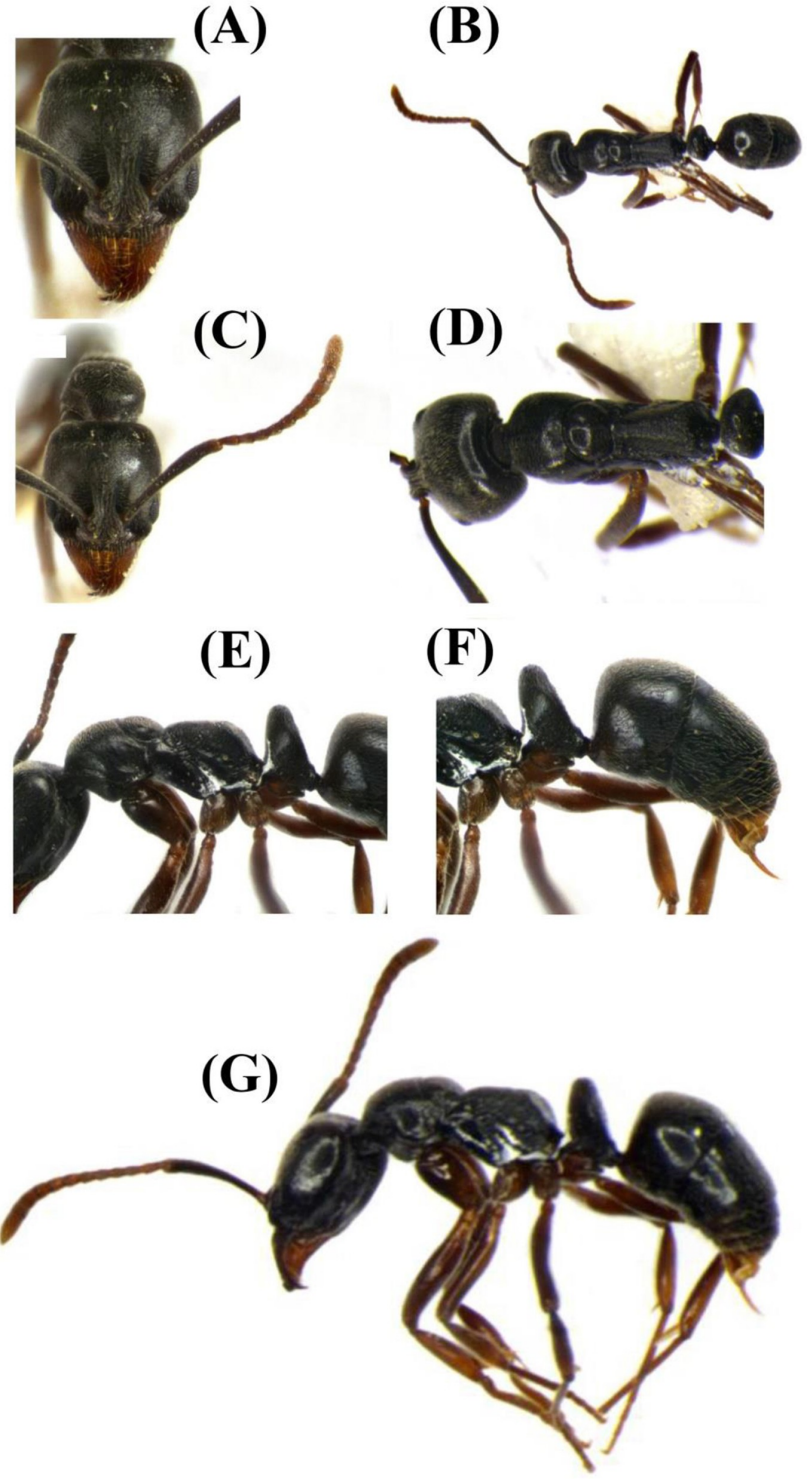
*Brachyponera nigrita* (worker). (A) Head in full face view (B) Habitus dorsal view (C) Head with antennae (D) Mesosoma dorsal view (E) Mesosoma lateral view (F) Abdomen lateral aspect (G) Habitus in profile view.

#### Description (worker) head

Head in full-face view clearly longer than wide with posterior margin straight and feebly convex sides; mandibles with series of small-median tooth, apical one acute and longest; clypeus narrow and slightly emarginated posteriorly; eyes of small size, maximum diameter (0.16 mm); head in full-face view eyes located below the mid length of head and just parallel to antennal insertion; antennal scape hardly reaching posterior head margin. **Mesosoma.** Promesonotal outline slightly indented; mesonotal grove distinct; propodeal spiracles rounded-ovoid; propodeal dorsum in profile making well defined declivity. **Sculpture.** Cephalic surface with minute punctations; clypeus with faint vestiges; mandibles with minute sculpture throughout; dorsum of pro-mesonotum smooth, metanotum weakly punctured; petiole slightly reticulate; gaster tergites smooth and shining but weakly reticulate in profile. **Pilosity.** Dorsum of head with small, white pubescent allover; antennae with abundant setae on funicular segment; clypeus with 4–5 long protrusive setae; gaster with scattered erect and suberect setae in profile. **Color.** Body uniformly dark brown-blackish.

#### Measurements (mm) of worker

Head length: 1.00, Head width: 0.93, Scape length: 0.94, Eye length: 0.2, Eye width: 0.16, Mesosoma length: 1.44, Pro-thorax width: 0.64, Petiole length: 0.36, Petiole height: 0.68, Petiole width: 0.55.

#### Distribution

India, Burma [34], Myanmar [51]; Pakistan (Present study)

#### Material examined

☿, Rawalpindi (Num Bhermal): N: 33.88225’ E: 73.35333’ 5122 elev.,03-04-2018, Under stone; ☿, Rawalpindi (Punjar): N: 33.63942’ E: 73.39592’ 2240 ft, elev., 05-05-2018, Under stone; ☿, Rawalpindi (Sang): N: 33.67568’ E: 73.41303’ 2803 ft, elev., 05-05-2018, Psyllid associated (Olive Tree); ☿, Rawalpindi (Kotli Sattian): N: 33.78607’ E: 73.51342’ 4158 ft, elev., 05-05-2018, Under stone; ☿, Rawalpindi (Kotli Sattian): N: 33.78702’ E: 73.51407’ 4174 ft, elev., 05-05-2018, Leaf litter; ☿, Rawalpindi (Num Bhermal): N: 33.89414’ E: 73.38242’ 1563 ft, elev., 29-04-2019, Under stone; ☿, Rawalpindi (Mallot): N: 33.87677’ E: 73.33968’ 4815 ft, elev., 16-09-2018, Tree trunk; ☿, Rawalpindi (Aryari): N: 33.75568’ E: 73.41775’ 4452 ft, elev., 29-12-2018, Under stone; ☿, Rawalpindi (Tarnosh): N: 33.55558’ E: 73.43732’ 15-03-2019, Under stone; ☿, Rawalpindi (Dhok ganj bari): N: 33.94089’ E: 73.40874’ 5946 ft, elev., 31-03-2019, Under stone; ☿, Rawalpindi (Lehtrar): N: 33.68697’ E: 73.42483’ 2917 ft, elev., 21-09-2018, Under stone; ☿, Rawalpindi (Num Bhermal): N: 33.87359’ E: 73.34788’ 15-07-2018, Aphid associated; ☿, Rawalpindi (Murree): N: 33.88769’ E: 73.4413’ 1804 ft, elev., 29-04-2019, dead wood; ☿, Rawalpindi (Shah Allah Ditta): N: 33.72035’ E: 72.91472’ 2069 ft, elev., 21-12-2018, Under stone; ☿, Rawalpindi (Phagwaari): N: 33.97948’ E: 73.48458’ 5032 ft, elev., 17-11-2018, Under stone; ☿, Rawalpindi (Gharial Camp): N: 33.91392’ E: 73.4387’ 5993 ft, elev., 17-11-2018, Under stone; ☿, Rawalpindi (Kuldhana): N: 33.92157’ E: 73.40188’ 6404 ft, elev., 15-07-2018, Aphid associated; ☿, Rawalpindi (Monal): N: 33.75022’ E: 73.05653’ 30-06-2019, Soil surface; ☿, Rawalpindi (Kaloyan): N: 33.6616’ E: 73.54643’ 676 m, elev., 20-07-2019, Under stone; ☿, Rawalpindi (Kuldhana): N: 33.92238’ E: 73.40268’ 6331 ft, elev., 03-04-2018, Grass; ☿, Rawalpindi (Tret): N: 33.83386’ E: 73.29692’ 3466 ft, elev., 16-09-2018, Aphid associated; ☿, Rawalpindi (Biya Chawan): N: 33.7434’ E: 73.4039’ 3960 ft, elev., 29-12-2018, Under stone; ☿, Rawalpindi (Moorii): N: 33.78863’ E: 73.42918’ 4615 ft, elev., 29-12-2018, Under stone; ☿, Rawalpindi (Murree): N: 33.9435’ E: 73.40428’ 31-03-2019, Under stone; ☿, Rawalpindi (Pir Sohawa): N: 33.78362’ E: 73.10982’ 4615 ft, elev., 30-06-2019, Tree trunk; ☿, Rawalpindi (Kaali Matti): N: 33.95646’ E: 73.39271’ 07-07-2019, Tree trunk; ☿, Rawalpindi (Khunda): N: 33.63538’ E: 73.49124’ 738 m, elev., 20-07-2019, Net sweep; ☿, Rawalpindi (Kotli Sattian): N: 33.81383’ E: 73.50242’ 1543 m, elev., 11-10-2019, Under stone.

### Nesting ecology (Fig 3)

**Fig 3:**
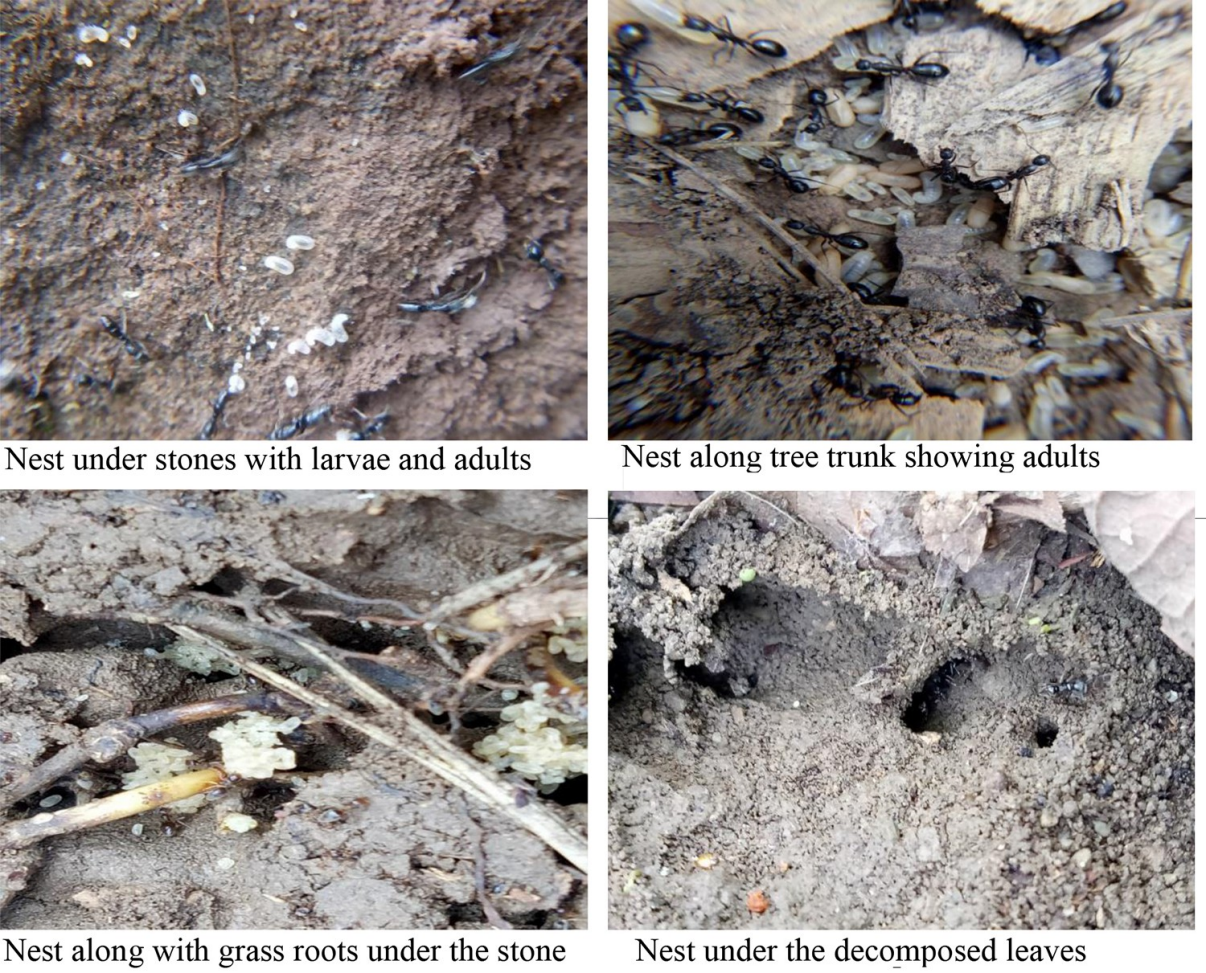
Illustration of nesting habitats of *Brachyponera nigrita*.

*B*. *nigrita* was collected from Northern and forest areas of Himalayan foothills of Pakistan, in Pothwar region. This species was collected from variety of habitats. Mostly its nests were found under the stones or under piles of decomposed leaves especially of pine leaves. They were found associated with collembolans in grass roots under the stone. One colony of these ants was observed under the stone with eggs, creamy white pupae along with isopods. Multiple nests under stone were also observed having plant debris in the nest. Termites were also present in some nests of these ants. Lepidopterous larvae have also been observed in a few nests. Many nests were present along the tree trunks under the bark of tree attacked with termite. Some nests were also found around the aphid colonies along the trunks of some plants. The collection sites were characterized by good amount of moisture, relative low temperature and dense pine forest.

### Maxent model results

#### Candidate models

As a whole, 1479 candidate models were run, with all combinations of 17 regularization multiplier settings, 29 feature class combinations, and 3 distinct sets of environmental variables. Model performance was evaluated based on statistical significance (Partial_ROC), omission rates (OR) and the Akaike information criterion corrected for small sample sizes (AICc)

**Fig 4:**
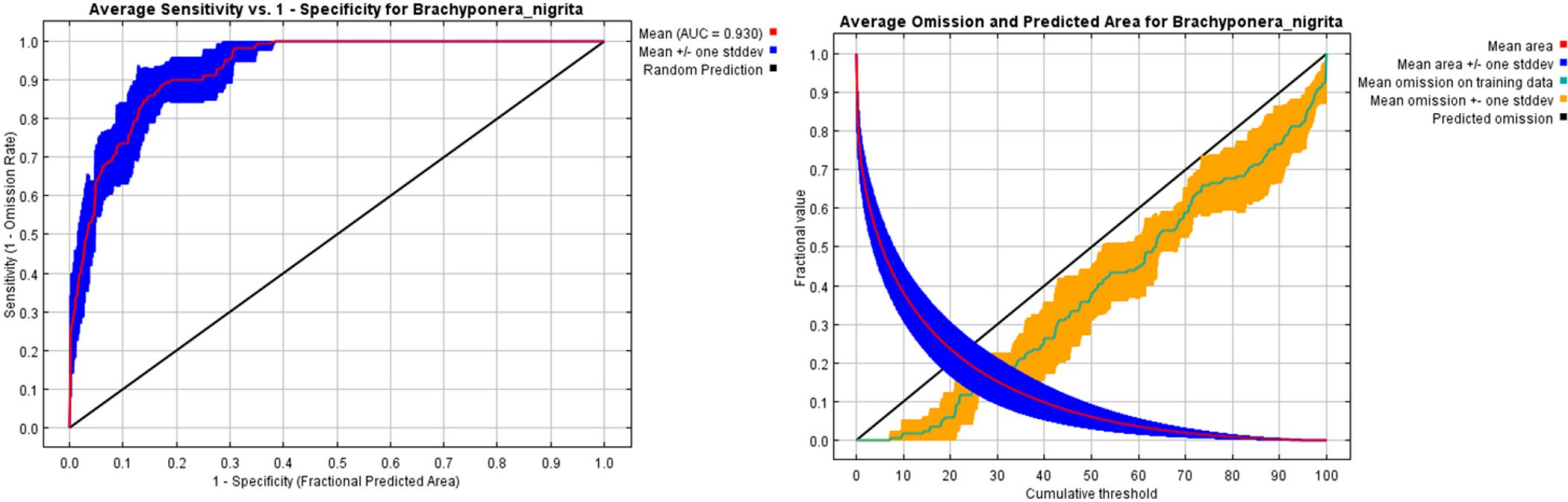
(a) Average sensitivity vs. 1- specificity for *Brachyponera nigrita*. (b) Average omission and predicted area for *Brachyponera nigrita*.

**Fig 5:**
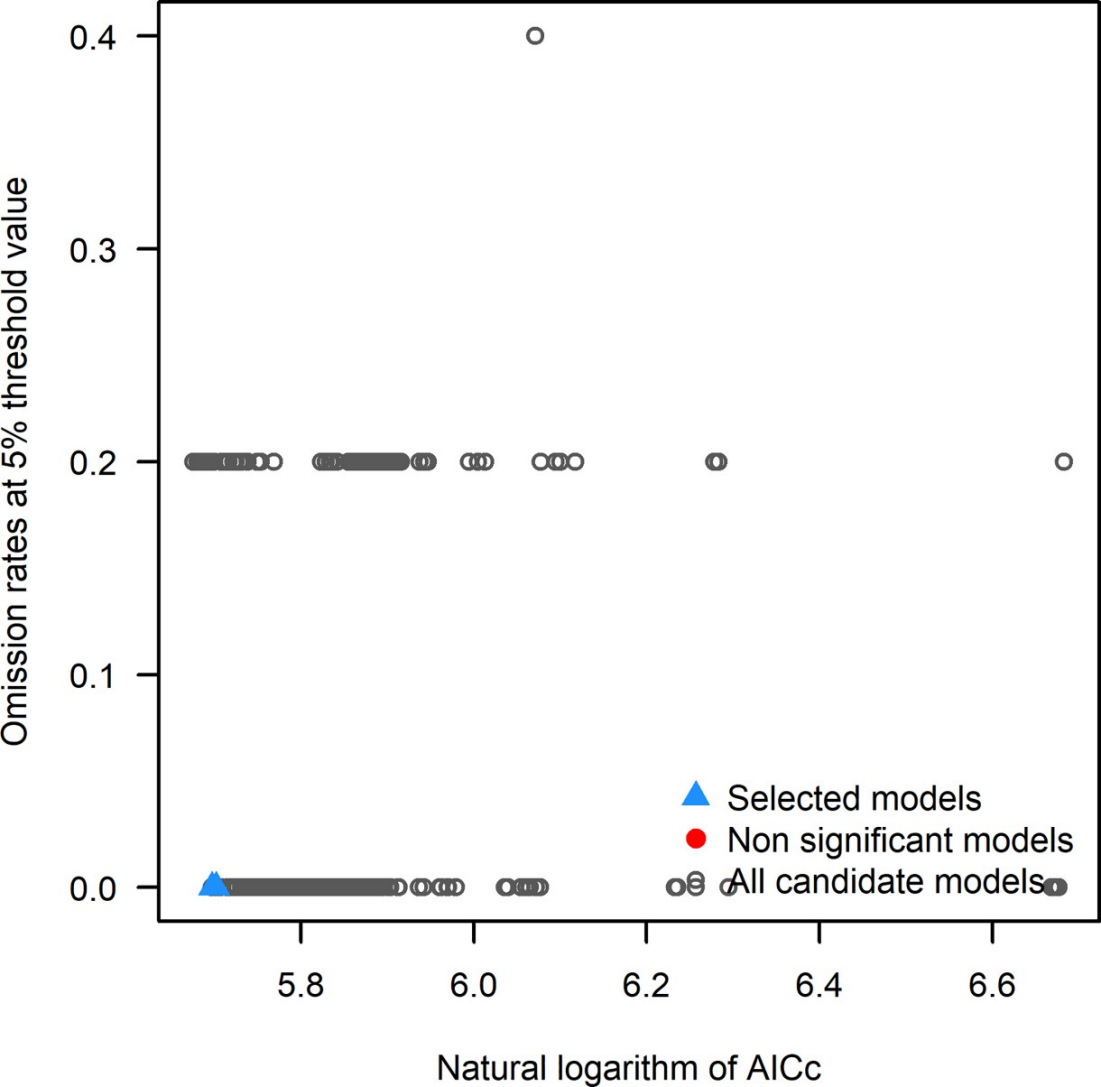
Distribution of all models in terms of the pre-defined criteria.

**Fig 6:**
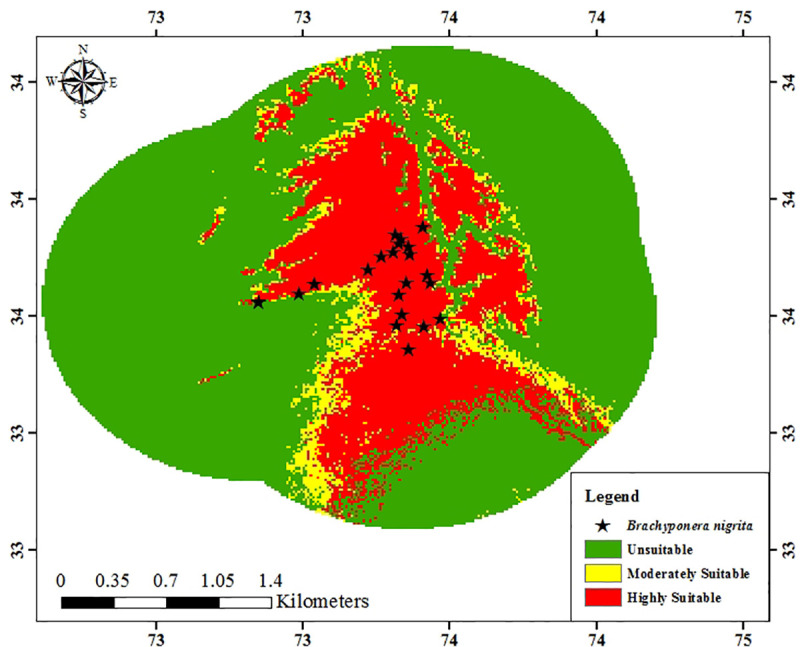
Predicted distribution of *Brachyponera nigrita* in calibration area by Maxent model.

[52] (Fig 4A and 4B). Among these only 1251 models were statistically significant and 2 were statistically significant models meeting omission rate and AICc criteria (Tables 2 and 3 and Fig 5). We used average of these two models for final model map preparation. Best model performance was determined at 5% threshold (Omission rates). Map of calibration area (Fig 6) predicted with maxent indicate that this species is currently distributed in Kashmir, Punjab, coastal areas of Sindh and Balochistan and a limited area of KPK provinces of Pakistan.

**Table 2:** General statistics of models that met distinct criteria.

Criteria	Number of models
All candidate models	1479
Statistically significant models	1251
Models meeting omission rate criteria	1015
Models meeting AICc criteria	3
Statistically significant models meeting omission rate criteria	787
Statistically significant models meeting AICc criteria	3
Statistically significant models meeting omission rate and AICc criteria	2

**Table 3:** Performance statistics for the best models selected based on pre-defined criteria.

Model	Mean AUC ratio	Partial ROC Omission rate at 5%	AICc	Delta AICc	W AICc	Number of parameters
**M_0.9_F_lq_Set3**	1.782	0	0298.140	0.000	0.673	4
**M_1_F_lq_Set3**	1.737	0	0299.485	1.344	0.258	4

#### Percentage contribution of predictor variables

**Fig 7:**
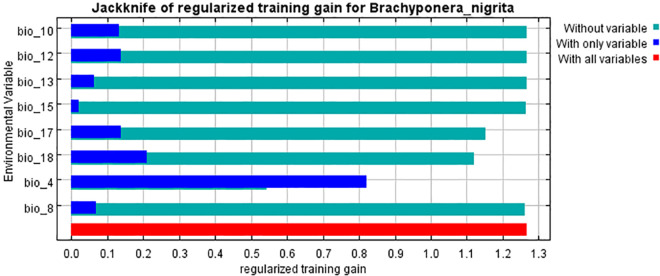
Jackknife of regularized training gain for *Brachyponera nigrita*.

The results of Jackknife test after running Maxent gave relative contribution of each bioclimatic variable for *B*. *nigrita* prediction (Fig 7). The main variables for predicting potential distribution of *B*. *nigrita* were Bio4 (Temperature seasonality), Bio18 (Precipitation of warmest quarter), Bio17 (Precipitation of driest quarter), Bio15 (Precipitation seasonality), Bio8 (Mean temperature of wettest quarter) and Bio12 (Annual precipitation) for the current geographical distribution with their contributions of 70.6%, 11.7%, 10.7%, 5%, 1.3% and 0.8% respectively (Table 4). Bio12 is the least contributing variable out of these variables. As a whole these four variables i-e (Bio4, Bio18, Bio17 and Bio15) contributed 98% for the prediction of potential distribution of the species as compared to all other variables. Bio4, Bio18 and Bio17 are also important for permutation (Table 4).

**Table 4:** Percentage contribution comparison of used environmental variables.

Variable	Units	Percent contribution	Permutation importance
Bio 4	Degrees Celsius	70.6	47.5
Bio 18	Millimeters	11.7	34.5
Bio 17	Millimeters	10.7	15.6
Bio 15	Fraction	5	0.5
Bio 8	Degrees Celsius	1.3	1.6
Bio 12	Millimeters	0.8	0.3
Bio 10	Degrees Celsius	0	0
Bio 13	Millimeters	0	0

**Bio4** (Temperature seasonality).

**Bio8 (**Mean Temperature of Wettest Quarter).

**Bio10** (Mean Temperature of Warmest Quarter).

**Bio12** (Annual Precipitation).

**Bio13 (**Precipitation of Wettest Month**)**.

**Bio15** (Precipitation seasonality).

**Bio17** (Precipitation of driest quarter).

**Bio18** (Precipitation of Warmest Quarter).

#### Model performance evaluation based on AUC values

When maximum entropy principle of Maxent is applied, it has been concluded that areas under receiver operating characteristic curves (AUC) test is 0.988 for current prediction and future projection [53, 54]. Our calculated AUC value shows that the results are significant (0.930) (Fig 4A). AUC value was considered as best and significant on the basis of knowledge based on previous studies [55, 56]

#### Current and future habitat suitability of *Brachyponera nigrita* in Pakistan

**Fig 8:**
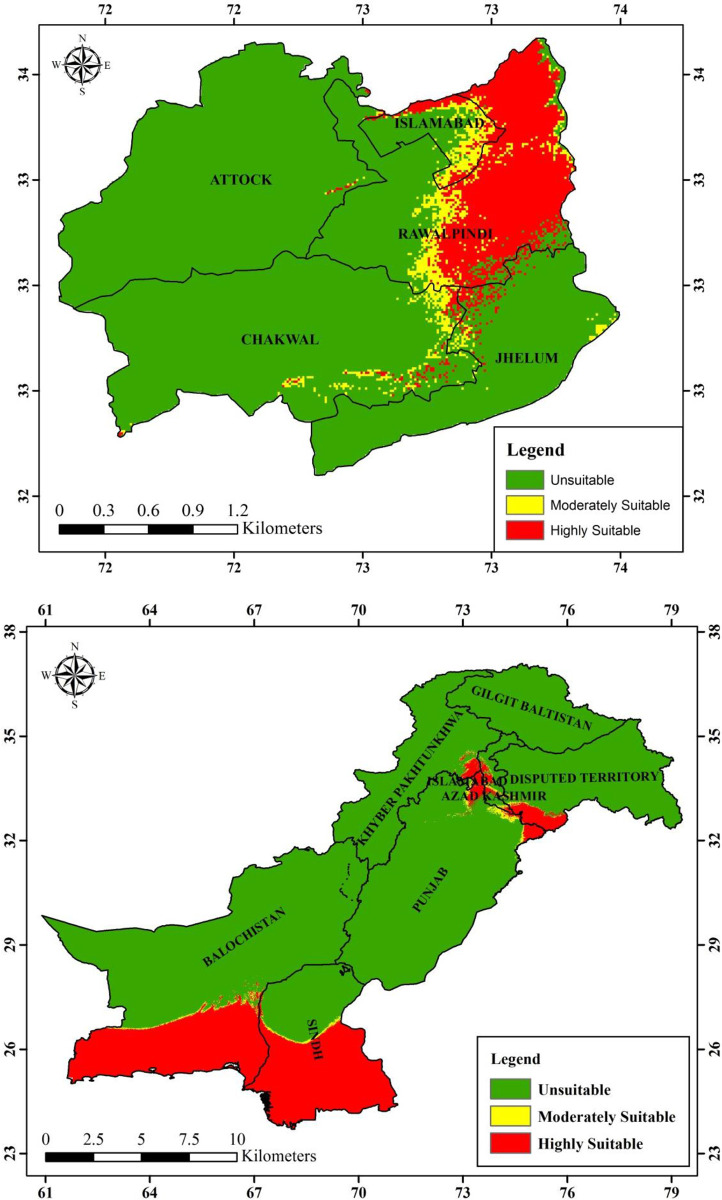
Current predicted distribution of *Brachyponera nigrita* by Maxent model.

**Fig 9:**
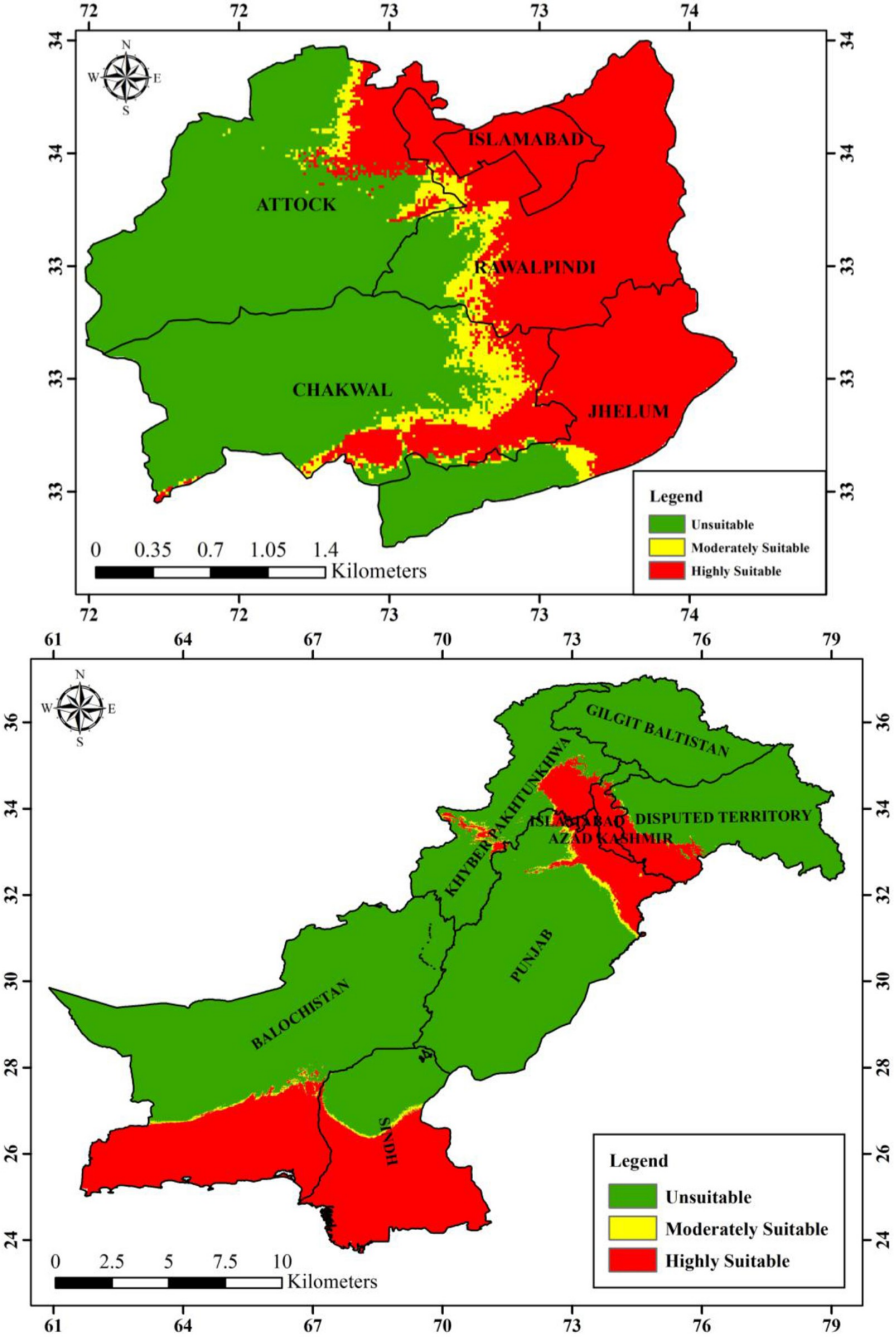
Future predicted distribution (RCP 4.5, 2050) of *Brachyponera nigrita* by Maxent model.

**Fig 10:**
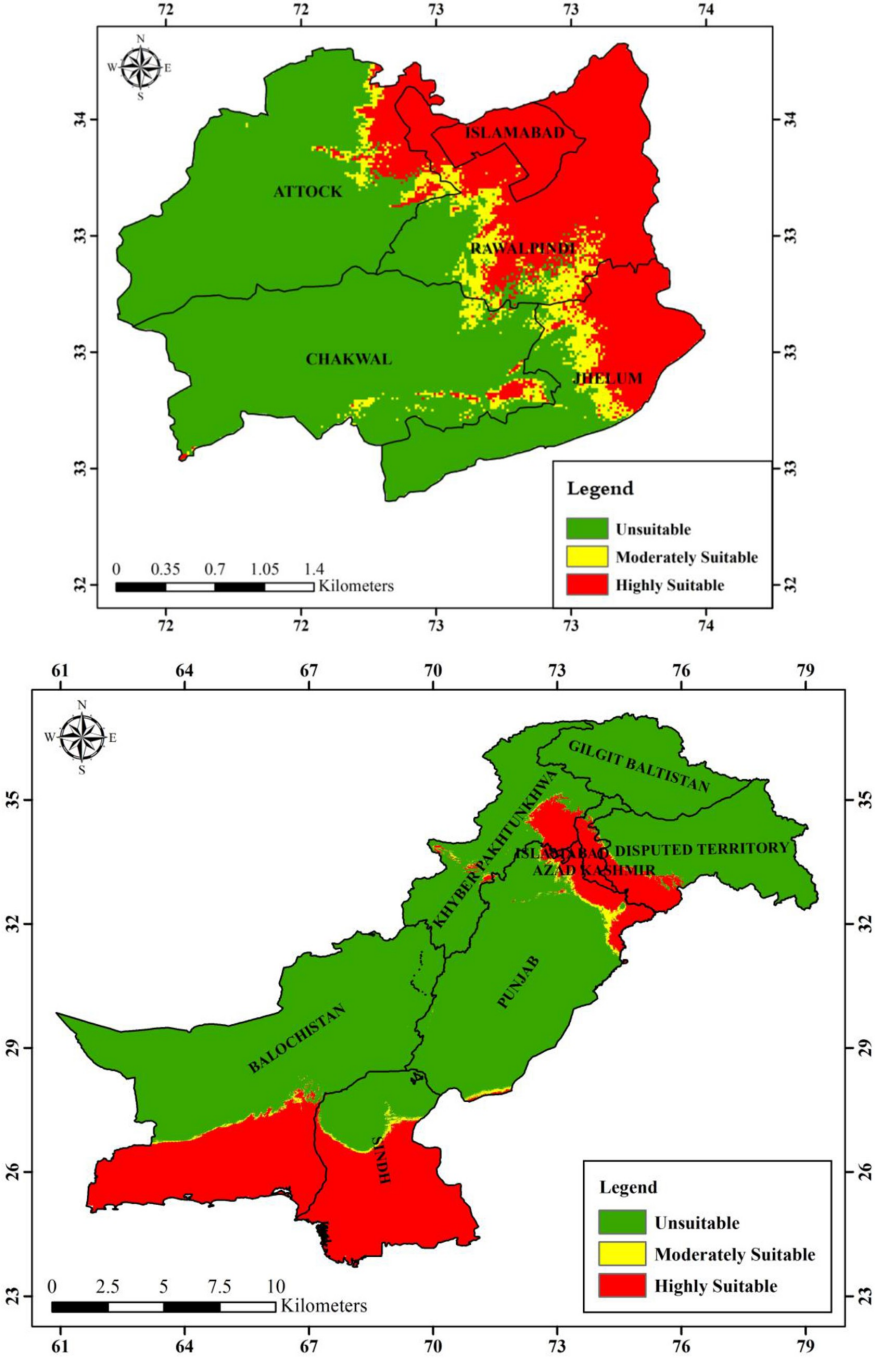
Future predicted distribution (RCP 8.5 2050) of *Brachyponera nigrita* by Maxent model.

The Maxent model provided the species future distribution under hypothetical projected climatic values for 2050 (RCP 4.5 and RCP 8.5) which were compared with bioclimatic values for current species distribution. The results (Figs 8–10) showed that averaged future predictions from bioclimatic Maxent model of 2050 (RCP 4.5) and (RCP 8.5), there would be an increase in moderately suitable area and highly suitable areas as compared to current distribution in Pakistan. In future this species is going to increase its suitable areas especially in KPK, AJK Punjab, Sindh and Balochistan. Its current predicted distribution is in some areas of Punjab, Kashmir, Sindh and Balochistan. This distribution is predicted to be with substantial increase in future in various areas of KPK, AJK Punjab, Sindh and Balochistan.

**Current and future hypothetical habitat suitability maps of Pothwar region.** The Maxent model provided the species future distribution under hypothetical projected climatic values for 2050 (RCP 4.5 and RCP 8.5) which were compared with bioclimatic values for current species distribution. The results (Table 5) showed that averaged future predictions from bioclimatic Maxent model of 2050 (RCP 4.5), there would be an increase in moderately suitable area by 505.932321km^2^ and increase in highly suitable areas by 6427.2576km^2^ as compared to current distribution. However under 2050 (RCP 8.5), there would be an increase of 572.118421km^2^ and 3765.140493km^2^ respectively in moderately suitable and highly suitable areas of *B*. *nigrita*.

**Table 5:** Comparison between current and future distribution area of *Brachyponera nigrita*.

Suitability Class	Threshold	Current and Future distribution area of *Brachyponera nigrita* (km^2^)					
		Current distribution	Future distribution RCP (4.5)	Change in area	Current distribution	Future distribution RCP (8.5)	Change in area
**Unsuitable**	**0–5%**	19313.450795	12380.145577	6933.305218	19313.450795	14977.976508	4335.474287
**Moderately Suitable**	**5–10%**	942.473448	1448.405769	-505.932321	942.473448	1514.591869	-572.118421
**Highly Suitable**	**>10%**	2973.178693	9400.436293	-6427.2576	2973.178693	6738.319186	-3765.140493

## Discussion

*Brachyponera nigrita* is distinct from *B*. *luteipes* and *B*. *jerdoni* in having longer joints of flagellum of antennae rather than broad [34]. Collected specimen were compared with published description of Bingham [21] and found similar. During collection, it was observed that workers were foraging at algae affected tree in forest. Schmidt and Shattuck [57] reviewed that Ponerine ants prefer to make their nests in soil, leaf litter or decaying wood. As it is clear from our results too that nests of *B*. *nigrita* were observed from same type of habitats. Additionally, our studies indicates that this species have some sort of association with other organisms like isopods, collembolan, termites etc. This indicates that this species may be predatory in nature too as discussed by Schmidt and Shattuck [57]. Its nesting habit in soil show that it likes low temperature and high humidity, which is also evident from our results of modeling too. It will move to low temperature areas in future.

Results of our model showed that geographical distribution of the species is dominantly influenced by four bioclimatic variables namely Bio4, Bio18, Bio17 and Bio15. Bio4 and Bio18 are also important for permutation. From these results of Maxent model, it can be concluded that *B*. *nigrita* would be increased under the influence of future climate change. Maxent results for current species distribution map reveal that *B*. *nigrita* is predicted to be increased in its distribution pattern in future under future predicted bioclimatics 2050 (RCP 4.5 and RCP8.5) in Pothwar region (Figs 8–10). Currently its distribution is limited in Attock and Chakwal region but in future its suitable areas are going to be increased as a whole in both districts in addition to increase in Rawalpindi and Islamabad. Highly suitable area of this species will also increase in Jhelum district of Pothwar region. Our studies are in line with other studies done in various parts of the world on ant’s distribution. It has been proved that ant’s distribution is largely limited by climate so is considered as most important factor globally affecting their distribution [58–60]. In various studies, species distribution models have been used to evaluate the potential impacts of future climate on habitat suitability and ant’s distribution. Kwon et al. [61] modeled the future distribution range of different ant species using generalized additive models and described that 12 species would shrink their suitable habitats, whereas five species would expand their distribution. Bertelsmeier et al. [62] and Bertelsmeier et al. [63] modeled 15 of the most invasive ant species for future and found that 8 would experience decrease in suitable areas and 5 would expand their distribution under future climate. According to retrospective analysis done by Needleman et al. [43] fire ants are expected to have an expanded range distribution due to predicted warming trends.

## Conclusions

*B*. *nigrita* prefer to make nest in soil, under stones, under the bark of tree etc. It may have some association with other organisms living in its nest. White eggs, pupae and adults may be further studied for estimating its associations with other organisms in the nests. There is a need of lab based studies for determination of its ecological role in various habitats. It may be concluded from the future predicted maps by Maxent model, future climate change would influence the potential distribution of *B*. *nigrita*. There will be a total increase in highly suitable habitat of the species in various localities of Pothwar region as well as different provinces of Pakistan with future bioclimatics. Its increased distribution will ultimately affect the total ecology of different organisms in various habitats in Pakistan. So it is recommended that species distribution modeling and GIS tools should be used to predict the impacts of future climatic conditions on various organisms as they are being used worldwide to cope with changing climatic conditions.
